# Beyond the bare minimum: the case for revised physical activity guidelines and protein intake recommendations that maximise healthspan

**DOI:** 10.3389/fnut.2026.1853124

**Published:** 2026-06-17

**Authors:** Chris Macdonald

**Affiliations:** University of Cambridge, Cambridge, United Kingdom

**Keywords:** aging, dietary guidelines, healthspan, physical activity guidelines, protein intake, public health policy, resistance training

## Abstract

Current physical activity and protein intake guidelines are framed around minimum thresholds, aimed primarily at preventing deficiency rather than optimising long-term health and functional capacity. Emerging evidence demonstrates that higher volumes and intensities of exercise (notably combining aerobic and resistance training) dramatically reduce all-cause mortality, enhance cognitive function, improve immune resilience, and preserve psychological well-being. Similarly, protein intakes well above existing recommendations support strength, recovery, and quality of life across diverse populations, including older adults and pregnant women. Importantly, well-planned high-protein diets can be safe, ethical, and environmentally sustainable. This perspective paper argues for a shift from ‘bare minimum’ public health targets to guidelines that promote optimal healthspan, empowering individuals to remain strong, independent, and cognitively capable throughout life. The paper recommends that governments update physical activity and protein guidelines to reflect contemporary evidence, accompanied by clear, actionable communications to ensure public awareness and uptake. In doing so, society can prevent avoidable declines in physical and cognitive function, reduce chronic disease burden, and cultivate a culture that values resilience, independence, and lifelong vitality.

## Introduction

### Physical activity recommendation

Regular physical activity produces benefits across multiple physiological and psychological systems, extending far beyond the more well-known strength and endurance outcomes. Recent evidence indicates that physical activity enhances immune function and immunologic responsiveness, suggesting an important role in disease defence (1). Exercise is associated with significant reductions in depression and anxiety, with randomised controlled trials demonstrating its efficacy as a non-pharmacological treatment (2). Robust meta-analytic data also demonstrate that structured physical activity improves cognitive outcomes, including memory and executive function, and may counteract cognitive impairment in ageing and neurodevelopmental disorders (3). In addition to mental health and cognition, physical activity consistently enhances sleep quality, reduces fatigue, and supports psychological well-being through multiple mediators such as self-esteem (4). Collectively, these findings underscore physical activity as a multi-domain intervention with broad implications for improved healthspan.

In short, the scientific consensus is clear; physical activity provides multiple profound positive benefits, and when it comes to the recommended dose, any amount is good but more is better. Any physical activity greater than ~15 min a day is associated with reduced all-cause mortality (5, 6). And mortality steadily declines with increasing weekly activity up to multiple hours per day (5, 7), and even better if muscle strengthening activity (resistance training) is added (6). When both aerobic activity and resistance training are increased, individuals can obtain a ~ 40% reduction in all-cause mortality (6).

Loss of muscle is one of the most evident and problematic conditions in older populations (8). As noted by Chen et al., it is associated with increased risk of falls by 60% and increased fractures by over 80% (2023). It substantially increases frailty, disability, loss of independence, hospitalisation, and institutionalisation in older adults, making it a major contributor to nursing home admission and decreased quality of life (9). Resistance training is recommended as the “first-line” treatment (10).

When comparing the least active with the most active, the benefits are particularly profound. For example, low muscular strength is associated with a ~ 200% increase in all-cause mortality risk relative to high strength (11). Very low cardiorespiratory fitness is associated with a ~ 400% higher mortality risk compared with high cardiorespiratory fitness (12). In contrast, smoking is associated with a ~ 50% increase in mortality risk (13).

It is also important to note that more is more with regard to intensity. Vigorous physical activity appears to be substantially more potent, on a per-minute basis, than lower intensities. Analyses of more than 70,000 adults from the UK Biobank suggest that vigorous activity is roughly four times more effective than moderate activity at reducing all-cause mortality risk, and around eight times more effective for equivalent reductions in cardiovascular mortality (14).

The contrast becomes even more pronounced when comparing vigorous activity with light activity. More than an hour of light activity may be required to achieve a reduction in cancer mortality risk comparable to that associated with just one minute of vigorous activity (14).

It is also important to explicitly clarify that vigorous exercise should not only be recommended to young people, and it is also important to clarify that it is not too late to start (providing one eases into it). In previously sedentary middle-aged adults, two years of high-intensity exercise training reversed key structural features of cardiac ageing, yielding effect sizes comparable to age-related differences spanning approximately two decades (15). These findings suggest that, even when initiated later in life and after prolonged sedentary behaviour, sustained high-intensity exercise may, in practical terms, ‘rewind’ certain features of cardiac ageing by decades (15) [please note the importance of progressive loading and medical clearance before starting vigorous exercise for long-term sedentary individuals (e.g., to mitigate acute cardiac risks)].

Accordingly, when looking at the most recent datasets, the evidence is clear that more is better: more variation, more time, and more intensity. Therefore, a challenging and varied protocol should be recommended for those seeking optimal health outcomes.

Given the profound benefits, it is concerning that many populations fail to achieve even the minimum activity guidelines, for example, the majority of adults in the U. S. fail to achieve an average of ~20 min a day (16). Also, given the profound benefits of going beyond the guidelines, it is concerning to see a lack of motivation and ambition in recommendations from the UK’s National Health Service (NHS). NHS recommendations are framed around minimums (e.g., aim for at least 20 min of moderate exercise a day) (17). The recommendations are not supported by the best available data nor do they explain the broader benefits. They do not provide an optimised routine or even a vision of what the best protocol would be with upper limits.

### Protein recommendation: amount

Protein is an essential macronutrient required for the maintenance, repair, and growth of body tissues, including muscle, bone, and connective tissue. Adequate protein intake supports metabolic functions, immune health, and the synthesis of enzymes and hormones (18). Its importance is particularly pronounced in physically active individuals, as exercise induces muscle protein breakdown and increases the demand for amino acids to support muscle repair and adaptation (19). Accordingly, alongside recommendations of increasing physical activity, protein intake needs to be considered.

The current dietary recommendation for protein intake in the UK is 0.34 grams of protein per pound of body weight, per day (g/lb./day). This figure was initially presented by the UK’s Department of Health in 1991 (20). However, this was intended as a minimum maintenance level, based on sedentary lifestyles. The goal was to prevent deficiency, with the primary aim of maintaining nitrogen balance.

In a nitrogen balance test, you compare nitrogen intake (from protein) with nitrogen losses in urine, faeces, and sweat. If losses exceed intake, the body is in negative nitrogen balance, meaning it is breaking down more protein than it is building. If intake and losses are equal, the body is in balance. In short, it was focused on not losing minimum capacity rather than gaining greater functional ability. The goal was not gaining strength, maximising health, physical function, or quality of life. Furthermore, there is a clear flaw in the initial report which states that the Panel could find no proven benefit of protein intakes in excess of the recommendation (20). There have since been multiple studies that highlight many benefits of daily protein intakes significantly greater than the UK recommendation.

For individuals who are physically active and engage in resistance training, higher protein intakes are associated with greater gains in muscle mass and strength, with evidence supporting benefits from protein intake more than double the UK’s recommendation (e.g., 0.6–1.6 g/lb./day) (19, 21). Even when the starting point is significantly above the UK recommendation, the additional protein can make a profound difference: increasing protein from ~0.5 g/lb./day to ~0.7 g/lb./day significantly enhances muscle growth (~30%) and strength (~10%) (19). As noted by Bosse and Dixon (22), the lay recommendation to consume 1 g of protein per pound of body weight, per day, while resistance training aligns well with research that assesses optimal health outcomes.

In addition to resistance training, endurance athletes have elevated protein needs because prolonged aerobic exercise increases amino acid oxidation and muscle protein breakdown, heightening the risk of net loss of muscle tissue if intake is insufficient (23). A narrative review of recent evidence supports daily protein intakes substantially above sedentary minimums for endurance athletes to promote recovery, support training adaptations, and preserve lean tissue, with habitual intakes equivalent to ~0.8 g/lb./day and recommendations often translating to even higher amounts during intensive training periods (23). Moreover, systematic meta-analysis shows that protein supplementation during endurance training can improve key performance outcomes such as time to exhaustion (24).

Several studies also demonstrate the importance of increased protein intake for elderly individuals to help prevent sarcopenia (loss of strength and function, primarily age-related). Recent studies consistently show improved muscle mass composition and improved function for elderly individuals when protein intake was raised to ~double the UK’s recommendation (25–28).

There is also significant data to suggest that pregnant women generally require ~double the UK’s recommendation to meet metabolic needs and support fetal growth; ~0.6 g/lb./day is associated with better fetal growth and lower risk of small-for-gestational-age infants, stillbirths, and neonatal deaths (29–32).

Furthermore, evidence suggests that high-protein diets may be particularly effective at facilitating fat loss. Protein can help increase satiety (protein increases the release of satiety hormones and reduces ghrelin, helping people consume fewer calories naturally) and protein has a higher thermic effect and thus slightly increases daily energy expenditure (~20–30% of calories are used to digest protein compared with ~5–10% to digest carbs or ~0–3% to digest fat). Multiple studies have shown protein intake of two-to-three times higher than the UK’s recommendation has been beneficial for fat loss (33, 34).

And so, beyond the more well-known case for elite athletes, higher protein intake can significantly improve quality of life for a significantly broader population. Importantly, current evidence does not demonstrate clinically meaningful harm in healthy individuals (35–38) (however, it is important to note caution for those with pre-existing conditions, e.g., kidney disease).

It should also be noted that although earlier research papers have suggested that approximately 20 g of protein per meal is sufficient to maximise acute muscle protein synthesis (39), more recent evidence indicates that substantially higher protein intakes per meal (e.g., 30–60 g) can be utilised (40–42). For example, contemporary studies suggest 25 g per meal as a minimum for ageing populations (40, 43).

In summary, there is significant evidence to suggest that national and international recommendations should be reviewed to be more in line with contemporary research, and any perceived disbenefits should be weighed against the apparent benefits on healthspan. For example, it would be important to consider if any risks are greater than, for example, the risk of sarcopenia or neonatal deaths.

### Protein recommendation: sources

In a recent study, omnivore and vegan nutrient intake was compared, and although several positives were noted (e.g., lower GHG emissions, higher fibre), it was found that vegans were less likely to have protein intake that is in line with current recommendations (44). This finding is in agreement with an earlier systematic review of 48 studies that concluded that although there appeared to be positive outcomes from a vegan diet, protein intake fell below the recommended levels (45).

While there are several potential health benefits of a vegan diet and no perceived inherent increased risk of mortality, there are legitimate concerns (45, 46). For example, a recent Oxford study found that vegans had higher risks of fractures (47). The authors suggest that the higher risks might be partially explained by lower protein intake (47). Likewise, a meta-analysis revealed that “women reporting that they adhered to vegan diets during pregnancy had offspring with lower mean birthweight and higher risk of preeclampsia (a serious pregnancy complication) compared with omnivorous mothers” (48). The authors suggested that low protein intake might be one plausible explanation for the observation (48).

In short, while there is evidence of multiple health benefits that come from following a plant-based diet, there is evidence to suggest that it often results in a lower protein intake, and that this may result in significant health risks. Furthermore, depending on the protein sources, there is evidence to suggest that one may need to consume a higher quantity of vegan protein sources to achieve the equivalent muscle building effects that come from some animal sources (e.g., milk protein vs. wheat protein) (49). However, well-balanced vegan meals that include complete proteins such as tofu or thoughtfully combined blends (e.g., fortified plant-based meal replacements, protein shakes, and supplementations) can provide all essential amino acids in sufficient high-quality amounts and provide equivalent beneficial effects (50–53).

In addition to the source of protein, it is also important to consider the ‘protein wrapper’ (the broader nutritional package in which the protein is delivered). For example, a high-sugar, highly processed protein bar may contain good quality and quantity of protein, but it also comes with added sugars, refined carbohydrates, and ultra-processed ingredients that may have adverse metabolic effects when consumed regularly. Accordingly, it will be important for consumers to not limit their selection criteria to mere protein quantity and quality. Fortunately, high-protein diets do not need to be inextricably linked to high-calorie diets. Clean ‘protein wrappers’ are widely available (e.g., tofu and chicken); therefore, a win-win (high-protein, low-calorie) can be achieved with considered meal choices. In fact, it is important to remember that, if managed correctly, high-protein diets can actively mitigate obesity via compounding mechanisms (e.g., the aforementioned satiety and thermic effect). High-protein groups lose more fat compared to low-protein groups with the same total calorie count; furthermore, high-protein groups observe a greater muscle protecting effect (54–57).

### Author recommendations

1. Governments to commission a review and update of exercise guidelines for optimal health outcomes.
2. Governments to commission a review and update of protein intake guidelines for optimal health outcomes.
3. Governments to create and deploy communications to ensure awareness and uptake of the updated guidelines.

With recommendations 1 and 2, note the term “optimal health outcomes”. Accordingly, the new recommendations ought to include what would be the best thing to do. Thus it will mean a move away from the current “bare minimum” framing. More broadly, this could be said to involve a cultural reframing as high-intensity exercise and high-protein diets are often associated with bodybuilders and superficial aesthetic goals. However, high-intensity exercise and high-protein diets also empower the general population to extend their lifespan and healthspan. Therefore, it is less about having “abs” and a “beach body” and more about being able to lift up, play with, and even remember, your grandchildren thanks to a strong and resilient body and mind.

When we see a stereotypical image of a hunched-over slow, fragile person with ill health, in their later years, it seems like an inevitable consequence of “Father Time”, however, I propose that in most cases, it is evidence of a non-evidence-based lifestyle. In short, we should not be quick to normalise and accept the consequences of a largely sedentary lifestyle; we should proactively empower people to reclaim their health and their independence. The reduction in unnecessary suffering would be profound. The UK and other governments should be ambitious and aspire to have the healthiest populations possible. Limiting recommendations to casual strolling and encouraging people to sit less, and reducing success to the number of daily steps is unambitious and inadequate. In my opinion, we should instead promote a culture that values strength, fitness, and purposeful movement across the lifespan, enabling people not merely to live longer, but to remain capable, independent, and vibrant throughout their lives.

With recommendation 3, note the term “communications to ensure awareness and uptake”. As a result, recommendations should be communicated in such a fashion that they are easy to understand and implement safely and effectively.

**Figure 1:**
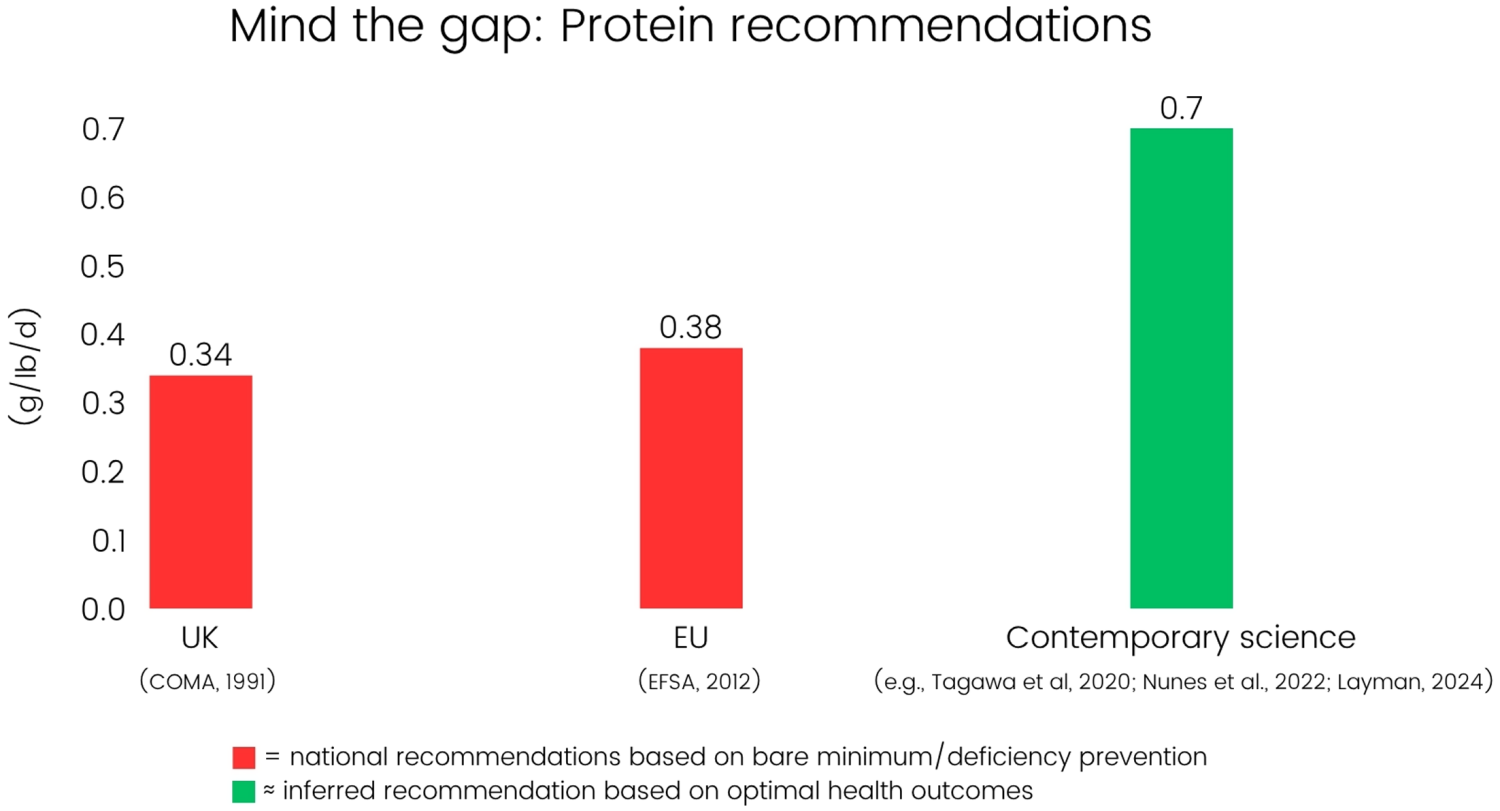
Bar graph showing protein recommendations, national recommendations versus contemporary science.

For example, based on the evidence explored in this paper, it is reasonable to suggest that the recommendation for optimal health outcomes for healthy and active adults could be in the range of 0.7 grams of protein per pound of target body weight, per day (as seen in Figure 1). Displayed on its own, that is not immediately clear or actionable. However, when communicated as a per meal target, it is much easier to put into action. For example, the average body weight of an adult in the UK is around 175 lbs. Based on that weight, the daily protein goal would be around 120 grams of protein, which would be an average of 30 grams per meal over four meals a day (noting that there is great flexibility with timings and doses as long as you broadly hit the target total amount). To make it easier still, the NHS could add a simple calculator app to their website, where you enter basic information (e.g., age, lifestyle, number of meals per day, goals) and it gives you the target per meal.

If, for example, you wanted to maximise muscle growth or you are older, the recommendation may well be at or above 1 (g/lb./day). Such a calculator could also more accurately indicate risks as entering a sub-optimal value in a given category could prompt evidence-based guidance.

Accordingly, in most cases, members of the public would be made aware that, for optimal health outcomes, they should be increasing cardio and resistance training, and therefore, they should be increasing protein intake. It should prioritise exercise and then the protein should be viewed as a way to support that. More precisely, not only exercise but targeted training for optimal health outcomes.

The patient should leave the calculator feeling more empowered and armed with concrete, actionable goals with the option for automated accountability check-ins.

Given the evolving and expanding nature of nutrition science, I would also recommend that future recommendations are viewed as an iterative and dynamic set of guidelines that are quickly updated based on the best available data.

## Conclusion

Dietary and lifestyle research is inherently complex, often relying on self-reported data, limited sample sizes, and study designs that make it challenging to fully account for confounding variables. For instance, an observed association between higher protein intake and adverse health outcomes may reflect correlated factors (such as lower consumption of fruits and vegetables or higher overall caloric intake) rather than protein intake per se. More concretely, epidemiological studies have reported that individuals adhering to omnivorous diets are, on average, more likely to smoke, consume alcohol, and have lower educational attainment compared with vegetarian or vegan groups, underscoring the need to carefully account for socioeconomic and behavioural confounders when interpreting diet-disease associations (58, 59). Furthermore, when specific diets or lifestyle patterns are adopted by only a small proportion of the population, the resulting scarcity of robust data constrains the strength and generalisability of conclusions.

Similarly, popular measurements such as caloric expenditure and oxygen consumption are very simple tools; we now have significantly more sophisticated tools that provide a more complete picture of the related health outcomes. More broadly, there needs to be a clearer acknowledgement of broader healthspan targets; exercise is all too often linked with the goal of fat loss, and therefore can seem unnecessary to those who either are not concerned about their body composition or are not motivated to lose weight. However, exercise is important for ageing better. It is about being more capable and independent for longer. And in an era where many nations are feeling the strain of an ageing population, this should be more front and centre of messaging.

It is also important to understand and acknowledge the limitations of broader terms: two people following a “vegan diet” could be eating meals that vary significantly with regard to nutrient profiles. Accordingly, it is important to remember that, for example, vegan diets per se are not inherently low in protein, as high-protein vegan foods could easily be incorporated into a vegan meal (e.g., tofu), likewise, omnivorous diets are not inherently low in fibre, as high fibre foods could easily be incorporated into an omnivorous meal (e.g., chicken with brown rice, artichoke, spinach, and broccoli).

Embracing the limitations and inconvenient truths will be essential for the progress of this field. We have to acknowledge that the quality and quantity of evidence is relatively low, and there is misleading reporting across the board. To truly understand exact amounts of protein for anabolic optimisation we would need a great deal more studies with larger sample sizes and greater dose variations.

This paper argues for revised guidelines, aligning them with the most recent evidence to achieve optimal health outcomes, and calls for continued exploration and updates. Preliminary analysis indicates that such updates would likely lead to recommendations for significantly increased volume, intensity, and variation of physical activity and significantly increased protein consumption. The paper noted that any perceived trade-offs would need to be considered in relation to the profound benefits on healthspan. The paper also found that there appears to be no inherent ethical trade-offs in the increase of protein intake. Adequate quantities and quality of protein sources can be found in meat-free meals, if carefully considered. Accordingly, meals can be optimised in such a way that they are profoundly more beneficial for both people and the planet.

It could be said that there is an underlying theme of this paper about questioning what it means to be ‘extreme’. For example, under modern standards, an elderly person running and weightlifting every day could be labelled as extreme, but are they in fact simply following the best available science and preventing avoidable suffering? Similarly, someone who follows a meat-free diet is often labelled as extreme, but are they also simply following the best available science and preventing avoidable suffering?

In the modern food system, approximately 80 billion land animals are slaughtered annually for meat production (60), and the majority are raised in intensive, factory-farming systems (61). At the same time, sedentary lifestyles (often characterised by approximately 8 hours of daily screen time) have become socially normalised. Practices that are widespread are rarely described as extreme, even when their long-term consequences are.

History suggests that social norms are not reliable indicators of rationality. Behaviours that are commonplace in one era are frequently regarded as excessive, harmful, or ethically troubling in retrospect. It is therefore plausible that future generations may view both industrial-scale animal production and chronically sedentary lifestyles as characteristic harmful excesses of our time.

Ultimately, the relevant question is not whether a lifestyle appears extreme relative to prevailing norms, but whether it is the best choice in light of the best available evidence. As scientific understanding advances, public health guidance and cultural expectations should evolve accordingly. If optimal dietary and exercise practices challenge established habits, this may reflect the inertia of tradition rather than extremism on the part of the early adopters.

## Data Availability

The original contributions presented in the study are included in the article/supplementary material, further inquiries can be directed to the corresponding author.
